# ITIH5, a p53-responsive gene, inhibits the growth and metastasis of melanoma cells by downregulating the transcriptional activity of KLF4

**DOI:** 10.1038/s41419-021-03707-7

**Published:** 2021-05-02

**Authors:** Jia Liu, Feng Cao, Xiaojie Li, Li Zhang, Zhengrong Liu, Xiaodong Li, Jingrong Lin, Chuanchun Han

**Affiliations:** 1 Department of Gynecology, Cancer Hospital of China Medical University, Liaoning Cancer Hospital & Institute, Shenyang, China; 2 Institute of Cancer Stem Cell & Department of Dermatology, The First Affiliated Hospital of Dalian Medical University, Dalian, 116044 China; 3 College of Stomatology, Dalian Medical University, Dalian, 116044 China; 4 Laboratory of Pathogenic Biology, College of Basic Medical Science of Dalian Medical University, Dalian, 116044 China; 5 Department of Gastrointestinal Surgery, People’s Hospital of China Medical University, People’s Hospital of Liaoning Province, Shenyang, China

**Keywords:** Skin cancer, Skin cancer

## Abstract

ITIH5, a member of the inter-α-trypsin inhibitory (ITI) gene family, acts as a putative tumour-suppressor gene in many cancers. However, its role and the regulatory mechanism in melanoma are still unclear. Here, we found that the expression of ITIH5 was decreased in melanoma tissues compared with normal skin tissues. Decreased expression of ITIH5 was correlated with clinicopathological features and predicted poor prognosis in patients with melanoma. Forced expression of ITIH5 significantly inhibited melanoma cell proliferation and metastasis in vitro and ex vivo while knockdown of ITIH5 expression enhanced the malignant behaviour of melanoma cells. In further mechanistic studies, we showed that p53 can directly bind to the promoter of ITIH5 and thus promotes transcription of ITIH5 in melanoma cells. Additionally, we found that ITIH5 interacted with Krüppel-like factor 4 (KLF4) and inhibited its transcriptional activity. Collectively, our data not only identified a tumour-suppressive role of ITIH5 in melanoma but also revealed that upregulation of ITIH5 by p53 suppressed melanoma cell growth and migration likely by downmodulating the transcriptional activity of KLF4.

## Introduction

Melanoma is the most aggressive skin cancer and has a high mortality rate^1^. Recently, a variety of treatment strategies, such as surgery, chemotherapy, targeted therapies (BRAF inhibitors or MEK inhibitors), immunotherapies (anti-CTLA4 or anti-PD1) and oncolytic viral therapy, have been used to improve the 5-year survival rate of patients with melanoma^2,3^. However, distant metastasis is still a major obstacle for treating melanoma. Thus, delineating the potential molecular mechanisms of tumour metastasis is important for improving the general prognosis of melanoma patients.

Metastasis is a multistep process in which tumour cells are released from a primary site to a different part or multiple parts of the body^4^. Accumulating evidence shows that turnover of the extracellular matrix (ECM) is a key step in driving the metastasis of various cancers^5,6^. Inter-α-trypsin inhibitory (ITI) proteins include a series of secreted serine protease inhibitors that are present in and stabilize the ECM^7,8^. Inter-alpha-trypsin inhibitor heavy chain 5 (ITIH5) is the fifth heavy chain member of the ITI family and is considered to be an important ECM modulator^9^. Under normal circumstances, ITIH5 is highly expressed in the placenta and moderately expressed in various organs. The dysfunction of ITIH5 has been shown to contribute to inflammatory dermatosis^10,11^ and obesity^12^. In the development of tumours, downregulation of ITIH5 expression caused by abnormal DNA hypermethylation has been reported in several types of cancers, including breast cancer^13^, bladder cancer^14^, colon cancer^15^, acute myeloid leukaemia^16^ and lung cancer^17^.

Accumulating evidence indicates that loss of ITIH5 is related to poor prognosis and malignant progression. In vitro, forced expression of ITIH5 suppressed cell growth and colony formation in the RT112 bladder^14^ and the CaSki cervical cancer cell lines^18^. In aggressive breast cancer, elevated ITIH5 expression induced epigenetic reprogramming and abrogated mesenchymal migration by upregulating the tumour-suppressor gene DAPK1 (ref. ^19^). In addition, overexpression of ITIH5 inhibited the invasive growth pattern of aggressive MDA-MB-231 breast cancer cells and modulated the expression of genes known to be involved in metastasis, including ENG, which led to a switch in TGF-β superfamily signalling^20^. In pancreatic ductal carcinoma, ITIH5 was identified as an important suppressor of metastasis by an unbiased RNA interference (RNAi) screen^21^. Recently, Young et al.^22^ have shown that an ITIH5 variant without secretion signal may suppress pancreatic cancer metastasis to the liver^22^. Although these data suggest that ITIH5 is a key tumour-suppressor gene in various cancers, its role and the regulatory mechanism are still unclear in melanoma.

In this study, we demonstrated that the mRNA and protein levels of ITIH5 were decreased in melanoma tissues compared with normal skin tissues. Loss of ITIH5 was related to the metastasis and progression of melanoma. Patients with low expression of ITIH5 had a poor prognosis. In vitro and ex vivo, enhanced ITIH5 expression significantly inhibited the growth and metastasis of melanoma cells. Further mechanistic investigations revealed that p53 directly bound to the promoter of ITIH5 and facilitated its transcription. We also identified ITIH5 as a bona fide interacting protein of KLF4 that suppressed the transcriptional activity of KLF4 and downregulated NUCB2, a known target gene of KLF4. In summary, our data identified a tumour-suppressive role of ITIH5 in melanoma, and revealed a new mechanism of p53 as a tumour suppressor in melanoma by transcriptionally activating ITIH5, which suppressed the transcriptional and oncogenic activities of KLF4.

## Materials and methods

### Cell culture and reagents

The human melanoma cell lines Mel-RM, Mel-CV, ME4405, A375 and Me1007 were cultured in Dulbecco’s modified Eagle’s medium (Gibco Invitrogen) containing 10% foetal bovine serum (ExCell Bio). Sk-Mel-28 and H1299 cells were cultured in Roswell Park Memorial Institute (RPMI) 1640 (Gibco Invitrogen) containing 10% foetal bovine serum (ExCell Bio). The following antibodies were used in this study: GAPDH (Santa Cruz Biotechnology, SC-25778, 1:1000), ITIH5 (Sigma, SAB1303414, 1:50), actin (Santa Cruz Biotechnology, sc-1616, 1:1000), KLF4 (Cell Signalling Technology, 12173 S, 1:500), NUCB2 (Abcam, ab229683, 1:300), Flag (Sigma, F3165, 1:1000) and GFP antibodies (Proteintech, 66002-1-Ig, 1:1000).

### Cell colony formation and migration assays

In the colony formation test, melanoma cells with overexpression or knockdown of indicated genes were diluted to a single-cell suspension, and 1000 or 2000 cells in each well of six-well plates were incubated with 5% CO_2_ at 37 °C for 10 days. The colonies were then stained with 0.04% crystal violet and 2% ethanol and manually quantified.

The melanoma cell migration assay was carried out in a 12-well Transwell plate with 8-mm polyethylene terephthalate membrane filters (Corning). Cells were allowed to migrate in the humidified chamber for 24 h. After the incubation period, the filter was removed, fixed with 4% formaldehyde for 15 min and stained with 0.1% crystal violet for 20 min, and the cells were counted.

### Ex vivo tumorigenesis and metastasis assays

Animal research was carried out according to the National Institute of Health Guide for the Care and Use of Laboratory Animals under the approval of the Animal Research Committee of Dalian Medical University. Male nude mice (4–6 weeks old, 18–20 g) were obtained from the SPF Laboratory Animal Center of Dalian Medical University (Dalian, China) and were randomly divided into the indicated groups. Indicated cells were subcutaneously injected into nude mice or through the lateral tail vein. After 6 days, the size of the tumour was measured by Vernier callipers every 2 days and converted to TV according to the following formula: TV (mm^3^) = (*a* × *b*^2^)/2, where *a* and *b* are the maximum and minimum diameters, respectively. All animals were euthanized 24 days after the injection, and the transplanted tumours were removed, weighed and divided into two for further study. The weights of the lungs and their metastatic nodules were measured in the mice injected through the lateral tail vein. The lungs were fixed with 4% formalin and embedded in paraffin blocks.

### Dual-luciferase reporter assay and chromatin immunoprecipitation assays

The promoters of ITIH5 as indicated were cloned into the basic vector pGL3, and the resulting plasmids were transfected into 293 T cells or melanoma cells. The luciferase activity was measured in a 1.5 ml Eppendorf tube, and according to the manufacturer’s recommendation after transfection, a Promega Dual-Luciferases Reporter Assay kit (Promega E1980) was used to determine luciferase activity. Relative Renilla luciferase activity was normalized to firefly luciferase activity.

Chromatin immunoprecipitation (ChIP) tests were performed using the Millipore ChIP kit (17-371RF) according to the manufacturer’s instructions. The reverse transcriptase PCR (RT-PCR) results of binding DNA fragments with specific primers are as follows: P5 Forward: 5′-TTCTCGCGTCCTCTGGCGAC-3′; Reverse: 5′-TTCCCCCCACCTCCCCAGTC-3′.

### Lentiviral shRNA constructs, packaging and infection

For generation of lentiviral shRNA constructs against human ITIH5 and p53, plasmids for shRNA ITIH5 were purchased from GE (Catalogue Number: RHS6066-200219251; 200161145; 200209532). The targeting p53 sequence was cloned into the pLKO.1-puro vector. The targeting sequences were as follows: No. 1 5′-CGGCGCACAGAGGAAGAGAA-3′; No. 2 5′-TCAGACCTATGGAAACTACTT-3′.

### Reverse transcription quantitative real-time PCR (RT-qPCR) and RNA sequencing

Total RNA was isolated using TRIzol (Invitrogen). One microgram of total RNA was reversely transcribed to cDNA using the PrimeScriptTM RT reagent kit (TaKaRa, RR047A) according to the manufacturer’s instructions. The primers were as follows: ITIH5 Forward: 5′-TTCCCGTTATGCCTTCACTAC-3′; Reverse: 5′-TTTCGCCCTGATACACCTTG-3′; actin Forward: 5′-ACCTTCTACAATGAGCTGCG-3′; Reverse 5′-CTGGATGGCTACGTACATGG-3′.

For RNA sequencing analysis, total RNA extraction, library construction, sequencing and data analysis were carried out by Biomarker Technologies, Beijing, China.

### Immunohistochemistry and lung histology

Human melanoma tissue microarrays (TMAs) were purchased from Alenabio (Xi’an, China; Catalogue Number: OS-Ski01003, DC-Mel11011, DC-Mel01007, DC-Mel11005b). These TMAs contained 42 skin tissues and 92 melanoma tissues that originated from different organs, including the vulva (3 cases), rectum (10 cases), tongue (1 case), stomach (2 cases), mediastinum (1 case), parotid gland (1 case) and skin (74 cases). We used an ITIH5 antibody to perform immunohistochemical (IHC) staining on the same paraffin-embedded tissue blocks that were used for clinical diagnosis. Immunohistochemistry was performed using the avidin–biotin complex method (Vector Laboratories), including heat-induced antigen retrieval procedures. The slice escape rate of the skin tissues was 5%, and that of melanoma tissues was 13%.

For lung histology, mice were sacrificed by carbon dioxide asphyxiation. Lungs were dissected under a fluorescence stereoscope, fixed in 4% formaldehyde overnight, embedded in paraffin, sectioned at 6 mm and stained with hematoxylin and eosin for pathology.

### Plasmid transfection, immunoprecipitation and GST pulldown

Cells were transfected with the indicated plasmids using Lipofectamine 3000 reagent (Invitrogen) according to the manufacturer’s protocol. For immunoprecipitation, cells were lysed with 1% NP40 lysis buffer (50 mM Tris-HCl, pH 8.0, 150 mM NaCl, 1% NP40, 0.5% deoxycholate) together with protease-inhibitor cocktail (Biotool). Cell lysates were incubated with the indicated primary antibodies and protein A/G agarose beads (Santa Cruz) at 4 °C. The immunocomplexes were then washed twice with 200 µl of PBS. Both lysates and immunoprecipitates were examined using the indicated primary antibodies followed by detection with the related secondary antibody.

#### GST pulldown assay

GST-KLF4 or GST-tag was purified from *Escherichia coli* strain BL21 (Invitrogen) with GST agarose beads. GST or GST-KLF4 bound to GST agarose beads was incubated with Flag-ITIH5 purified from HEK293T cells for 6 h at 4 °C. The beads were then washed four times with PBS, followed by western blotting.

### Statistics and data analyses

The data are expressed as the mean ± SD, and were statistically evaluated using GraphPad Prism 5. Multiple comparisons between treatment groups and control groups were performed using Dunnett’s least significant difference (LSD) test. Statistical significance between groups was calculated using the LSD test in SPSS 17.0 software (SPSS, Inc., Chicago, IL, USA). Values of *p* < 0.05 were considered statistically significant.

## Results

### ITIH5 inhibits the growth and metastasis of melanoma cells

**Fig. 1:**
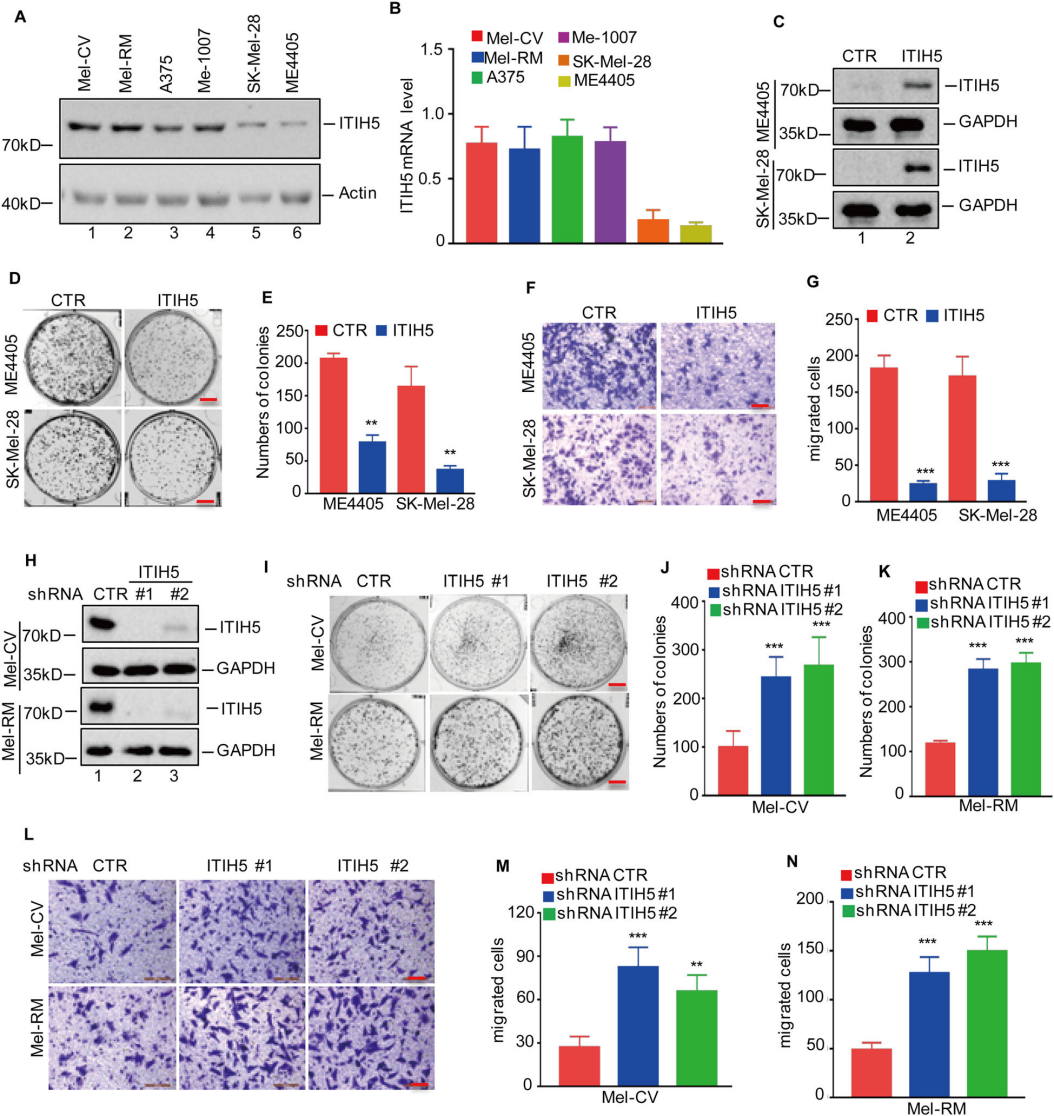
ITIH5 inhibits the growth and migration of melanoma cells in vitro. **A** Protein levels of ITIH5 in the indicated melanoma cell lines were evaluated by western blotting. Actin was used as the loading control. **B** ITIH5 mRNA levels in the indicated melanoma cell lines were assessed by RT-qPCR. Shown were means and standard deviations from three independent experiments. **p < 0.01, ***p < 0.001. **C** Protein levels of ITIH5 in ME4405 and SK-Mel-28 cells overexpressing ITIH5 were verified by western blotting. GAPDH was used as the loading control. **D**, **E** Representative images of culture plates (**D**) and average number of colonies (**E**) from colony formation assays. Data in **E** represent three independent experiments. **p < 0.01. Scale bar, 1 cm. **F**, **G** Represented images of crystal violet-stained culture plates (**F**) and average number of migrated cells (**G**) from Transwell cell migration assays. Data in **G** represent three independent experiments. ***p < 0.001. Scale bar, 100 μm. **H** Protein levels of ITIH5 in Mel-CV and Mel-RM cells with ITIH5 knockdown were verified by western blotting. GAPDH was used as the loading control. **I**–**K** Representative images of culture plates (**i**) and average numbers of colonies (**J**, **K**) from the colony formation assays. Data in **J**, K represent three independent experiments. ***p < 0.001. Scale bar, 1 cm. **L**–**N** Represented images of crystal violet-stained culture plates (**L**) and average number of migrated cells (**M**, **N**) from Transwell cell migration assays. Data in **M** and **N** represent three independent experiments. **p < 0.01, ***p < 0.001. Scale bar, 100 μm.

To evaluate the contribution of ITIH5 to the development of human melanoma, we first detected the expression levels of ITIH5 in a panel of six melanoma cell lines by western blotting and RT-qPCR. The results showed that both protein and mRNA levels of ITIH5 in SK-Mel-28 and ME4405 cells were generally lower than those in Mel-CV, Mel-RM, A375 and Me1007 cells (Fig. 1A, B). Thus, we overexpressed ITIH5 in SK-Mel-28 and ME4405 cells and verified the overexpression of ITIH5 by western blotting (Fig. 1C). Then, we investigated the effect of ITIH5 overexpression on cell proliferation by colony formation assays. As shown in Fig. 1D, E, increased ITIH5 expression significantly decreased the number of colonies as compared with that of the control group. In the Transwell cell migration assay, the extent of migration of ITIH5-overexpressing melanoma cells was remarkably lower than that of control cells (Fig. 1F, G). To further confirm the role of ITIH5 on decreasing cell proliferation and inhibiting cell migration, we knocked down ITIH5 expression with two independent shRNAs in Mel-CV and Mel-RM cells. As shown in Fig. 1H, compared with those of the control group, the expression levels of ITIH5 in the shRNA ITIH5 groups were substantially reduced. Then, we performed the colony formation assay to assess the effect of ITIH5 on cell growth, and found that compared with the control group, ITIH5 depletion promoted cell proliferation (Fig. 1I–K). Consistent with an inhibitory role of ITIH5 on cell migration, more ITIH5-depleted cells passed through the membrane than the control cells (Fig. 1L–N).

**Fig. 2:**
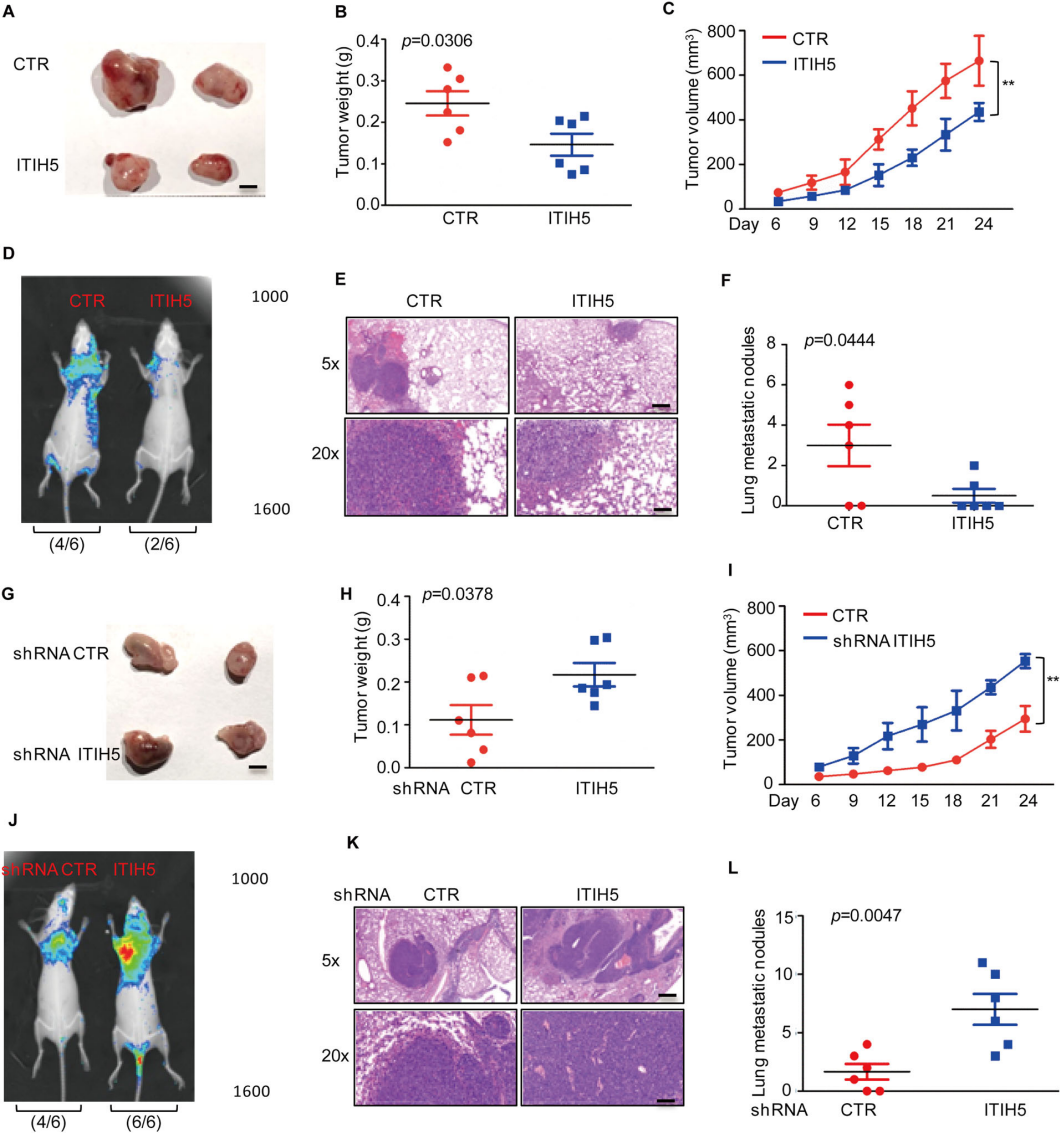
ITIH5 suppresses the growth and migration of melanoma cells ex vivo. **A**–**C** SK-Mel-28 cells with or without overexpression of ITIH5 were injected subcutaneously (1 × 10^7^ cells per mouse) into nude mice (*n* = 6). Represented images of xenograft tumours (**A**). The weight (**B**) and volume (**C**) of the tumours were calculated and analysed. Data in **B**, **C** were analysed by Student’s *t*-test, *p* = 0.0306, **p < 0.01. Scale bar, 0.5 cm. **D**–**F** ITIH5 was overexpressed in GFP-labelled SK-Mel-28 cells. The cells (5 × 10^6^ cells per mouse) were injected intravenously into nude mice (*n* = 6 per group). Two weeks later, the mice underwent bioluminescent imaging (**D**). Representative images of HE staining are displayed (**E**). Scale bars, 400 μm (top) and 100 μm (bottom). Each group of metastatic nodules was assessed (**F**), and data in **F** were analysed by Student’s *t*-test, *p* = 0.0444 **F**. **G**–**I** Mel-RM cells with or without ITIH5 knockdown were injected subcutaneously (1 × 10^7^ cells per mouse) into nude mice (*n* = 6). Represented images of xenograft tumours (**G**). The weight (**H**) and volume (**I**) of the tumours were calculated. Data in **H**, **I** were analysed by Student’s *t*-test, *p* = 0.0378, **p < 0.01. Scale bar, 0.5 cm. **J**–**L** ITIH5 was knocked down in the GFP-labelled Mel-RM cells. Cells (5 × 10^6^ cells per mouse) were injected intravenously into nude mice (*n* = 6). Two weeks later, the mice underwent bioluminescent imaging (**J**). Representative images of HE staining are displayed (**K**). Scale bars, 400 μm (top) and 100 μm (bottom). Metastatic nodules were assessed in each group (**L**). Data in **L** were analysed by Student’s *t*-test, *p* = 0.0047.

To better understand the role of ITIH5 in melanoma cells, we subsequently investigated the effects of ITIH5 on tumour growth and metastasis ex vivo. To this end, we first implanted SK-Mel-28 cells with or without ITIH5 overexpression in nude mice. ITIH5 overexpression significantly suppressed the growth of SK-Mel-28 xenograft tumours, as indicated by the reduced tumour weights and tumour sizes compared with those of the control group (Fig. 2A–C). Then, we investigated the effect of ITIH5 on lung metastasis using xenograft models. GFP-labelled SK-Mel-28 cells with or without ITIH5 overexpression were injected intravenously in nude mice, which were subjected to bioluminescent imaging (BLI). As shown in Fig. 2D through BLI monitoring, mice receiving cells overexpressing ITIH5 had significantly decreased fluorescent signals as compared with the control group, suggesting that ITIH5 inhibits tumour metastasis. Further histological analysis revealed a significant decrease in the number of metastatic lesions in mice receiving cells overexpressing ITIH5—than in the control cells (Fig. 2E, F). In contrast, loss of ITIH5 in Mel-RM cells promoted tumour progression and lung metastasis ex vivo (Fig. 2G–L). Overall, these data suggest that ITIH5 is a bona fide inhibitor of tumour growth and metastasis in melanoma.

### ITIH5 is downregulated in human melanoma

**Fig. 3:**
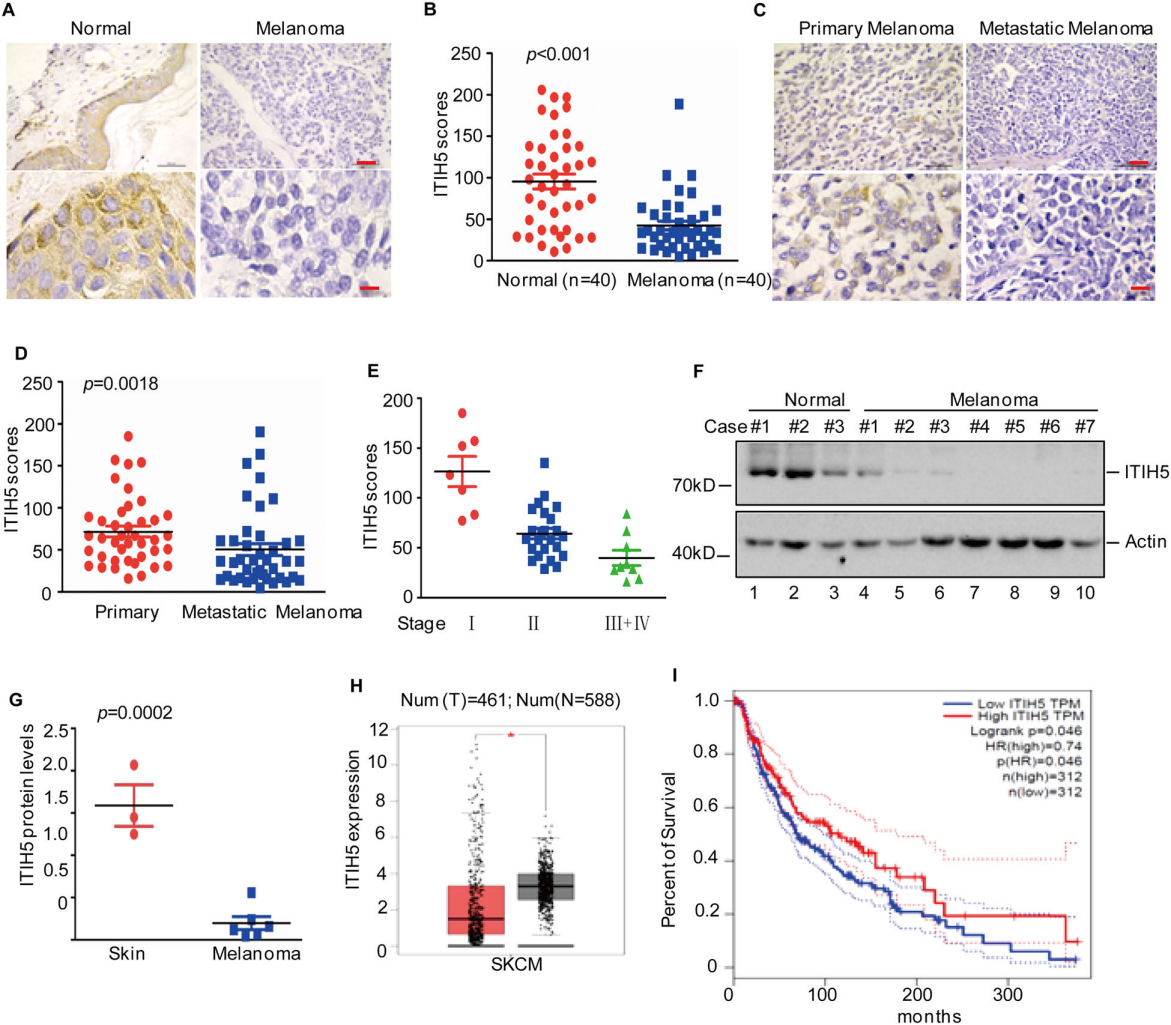
Low expression of ITIH5 in human melanoma tissues. **A** Representative microphotographs of ITIH5 immunohistochemical staining of normal skin tissues and melanoma tissue sections. Scale bar, 400 μm (up), 40 μm (down). **B** The expression of ITIH5 in normal skin tissues and melanoma tissues was assessed. The data were analysed by Student’s *t*-test, *p* < 0.001. **C** Representative microphotographs of ITIH5 immunohistochemical staining were performed in the indicated melanoma tissues. Scale bar, 400 μm (up), 40 μm (down). **D** The expression levels of ITIH5 in these tissues were quantified. The data were analysed by Student’s *t*-test, *p* = 0.0018. **E** The relationship between ITIH5 expression and tumour staging was analysed. The data were analysed by Student’s *t*-test, *p* < 0.001. **F**, **G** The expression of ITIH5 in fresh skin and melanoma tissues was analysed by western blotting (**F**). Actin was used as the loading control. The expression levels of ITIH5 were elevated (**G**) The data were analysed by Student’s *t*-test, *p* = 0.0002. **H** Analysis of ITIH5 expression in skin melanoma tissues and normal tissues. The data were obtained from the GEPIA database. The data were analysed by Student’s *t*-test, *p < 0.05. **I** Kaplan–Meier plot of the overall survival rate of 138 patients with melanoma. The data were obtained from the GEPIA database. Cut-off-high was set as 32% and cut-off-low was 68%.

To confirm the tumour-suppressive role of ITIH5 in melanoma, we first examined the expression of ITIH5 in 40 formalin-fixed and paraffin-embedded melanocytic tumours and 40 normal skin tissues by IHC staining of TMAs. We found that the expression of ITIH5 in melanoma tissues was significantly lower than that in normal skin tissues (Fig. 3A, B). To further evaluate the relationship between clinicopathologic variables and ITIH5 expression levels, we then utilized IHC to assess the expression levels of ITIH5 in primary and metastatic melanoma tissues. The IHC results showed that the expression of ITIH5 in metastatic melanoma was reduced as compared with that in primary melanoma (Fig. 3C, D). In addition, reduced ITIH5 expression was associated with the staging of melanoma (Fig. 3E). Consistent with data from the aforementioned TMA studies, a reduction in ITIH5 expression assessed by western blotting analysis was also evidenced in a panel of fresh melanoma tissues as compared with normal skin tissues (Fig. 3F, G). These findings were further validated through bioinformatics analysis of RNA-seq data of melanoma patients obtained from the GEPIA database as the mRNA levels of ITIH5 were lower in tumour tissues than in normal tissues (Fig. 3H). In addition, we also found that melanoma patients with relatively low ITIH5 levels showed lower survival rates than patients with high ITIH5 levels in the tumours (Fig. 3I). Taken together, these data strongly suggest that ITIH5 is an important tumour suppressor in melanoma whose expression levels are positively correlated with overall survival rates of melanoma patients.

### The tumour-suppressor gene p53 upregulates ITIH5 expression

**Fig. 4:**
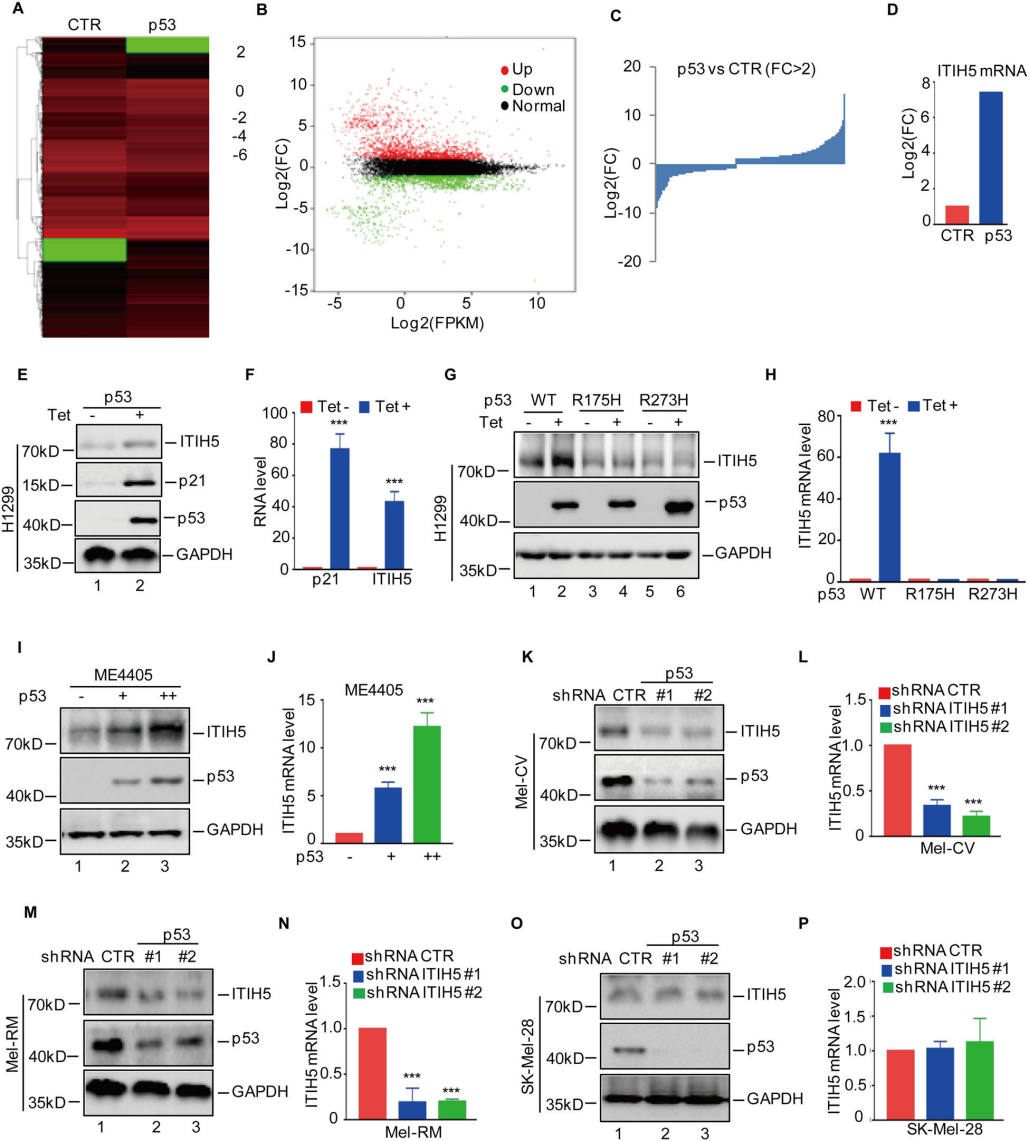
p53 upregulates ITIH5 expression. **A**–**C** H1299 cells expressing wild-type p53 induced by doxycycline were incubated with doxycycline for 24 h. Cell lysates were subjected to RNA sequencing analysis (**A**), and the altered genes were identified (**B**, **C**). **D** The change of ITIH5 mRNA level is shown. **E**, **F** Western blotting (**E**) and RT-qPCR (**F**) were used to detect the expression of ITIH5 in H1299 cells induced by doxycycline. GAPDH was used as the loading control. Data in **F** represent three independent experiments, ***p < 0.001. **G**, **H** Western blots (**G**) and RT-qPCR (**H**) were used to detect the expression of ITIH5 in the H1299 cells expressing wild-type p53 or mutant p53 induced by doxycycline. GAPDH was used as the loading control. Data in **H** represent three independent experiments, ***p < 0.001. **I**, **J** p53 was transfected into ME4405 cells as indicated. The expression level of ITIH5 was analysed by western blotting and qRT-PCR. GAPDH was used as the loading control. Data in **J** represent three independent experiments, ***p < 0.001. **K**–**P** ITIH5 was knocked down in Mel-CV (**K**), Mel-RM (**M**) and SK-Mel-28 (**O**) cells. The expression level of ITIH5 was analysed by western blotting and RT-qPCR. GAPDH was used as the loading control. Data in **L**, **N**, **P** represent three independent experiments, ***p < 0.001.

The gene that encodes for p53 often has inactivating mutations in many human cancers^23^. To identify p53 regulatory genes, we used a Tet-On regulatory system to establish stable expression of wild-type p53 in the p53-null H1299 cell line. The cells were then subjected to RNA sequencing analysis, and 1666 upregulated and 967 downregulated genes were obtained (Fig. 4A–C). Among the differentially expressed genes, ITIH5 was notably upregulated in the p53-overexpressing cells (Fig. 4D). This result was further verified by western blotting and RT-qPCR analysis as we found that ITIH5 expression was dramatically increased in response to p53 overexpression (Fig. 4E, F).

Subsequently, we extended our analysis by using two p53 mutant alleles (p53-R175H and p53-R273H), and confirmed the role of p53 on upregulating ITIH5. Specifically, we observed that although a wild-type p53 allele increased the expression of ITIH5, both mutant alleles failed to do so (Fig. 4G, H). To further prove that ITIH5 was a true target gene of p53 in melanoma, we transfected the wild-type p53 into ME4405 cells, which are a p53-null melanoma cell line. Ectopic expression of p53 elevated the protein and RNA levels of ITIH5 in a dose-dependent manner as compared with those of the control cells (Fig. 4I, J). Additionally, we knocked down p53 expression with two independent shRNAs in melanoma cells and found that depletion of wild-type p53 suppressed the expression of ITIH5 in Mel-RM cells (Fig. 4K, L). Similar results were obtained in Mel-CV cells (Fig. 4M, N). Importantly, downregulation of p53 did not change expression levels of ITIH5 in the p53-mutated SK-Mel-28 cells (Fig. 4O, P). All these data support that p53 upregulated the expression of ITIH5 in melanoma cells.

### p53 directly binds to the promoter of ITIH5

**Fig. 5:**
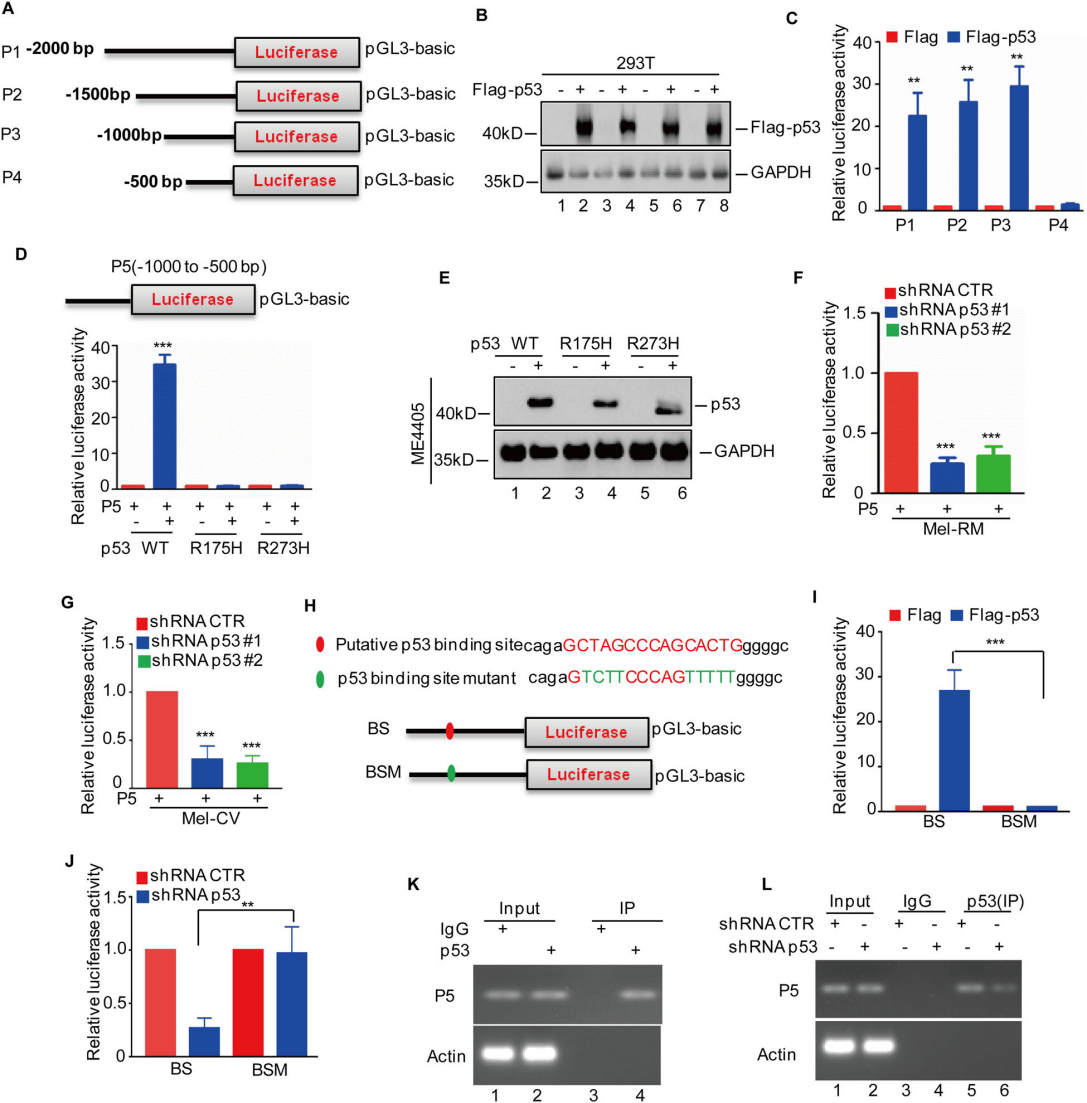
p53 directly binds to the promoter of ITIH5. **A** Schematic illustration of the pGL3-based reporter constructs used to detect the transcriptional activity of ITIH5 by luciferase assays. **B**, **C** Parts of the ITIH5 promoter, named P1, P2, P3 and P4, were transfected individually into 293 T cells with or without p53 overexpression. The expression levels of p53 were detected by western blotting (**B**). GAPDH was used as the loading control. The luciferase activity was measured (**C**). Data in **C** represent three independent experiments, **p < 0.01. **D**, **E** Schematic illustration of the key region of the ITIH5 promoter used for the pGL3-based reporter construct. Part of the ITIH5 promoter named P5 was transfected into ME4405 cells together with wild-type p53 or mutant p53. The luciferase activity was measured (**D**). The results represent three independent experiments, ***p < 0.001. The expression levels of p53 or mutants were detected by western blotting (**E**). GAPDH was used as the loading control. **F**, **G** P5 was transfected into Mel-RM (**F**) and Mel-CV (**G**) cells with or without p53 knockdown. The luciferase activity was measured. The results represent three independent experiments, ***p < 0.001. **H** Schematic illustration of the p53 wild-type binding site (BS, −770 to −755 bp) and the matching mutant (BSM) that were used in luciferase assays. **I** BS and BSM were transfected into HEK293T cells with or without p53 overexpression. The luciferase activity was measured. The results represent three independent experiments, ***p < 0.001. **J** BS and BSM were transfected into Mel-RM cells with or without p53 knockdown. The luciferase activity was measured. The results represent three independent experiments, ***p < 0.001. **K**, **L** ChIP analysis showed the binding of p53 to the promoter of ITIH5 in Mel-RM cells with or without p53 knockdown. An isotype-matched IgG was used as a negative control.

To identify the p53-binding regions on the ITIH5 promoter, we first cloned the upstream sequence of ITIH5 and different truncations by PCR. Then, we inserted them into the pGL3-based luciferase reporter plasmids, which were named P1–P4 (Fig. 5A). We then transfected them into 293 T cells with or without p53 overexpression. The protein levels of p53 were evaluated by western blotting (Fig. 5B). As shown in Fig. 5C, the luciferase activities of cells transfected with P1, P2 and P3 increased in the p53-overexpressing cells. However, such an increase was diminished when P4 was transfected, suggesting that this region (−1000 to −500 bp) was critical for p53 to elevate ITIH5 expression. To confirm this, we cloned the region from −1000 to −500 bp into the pGL3-based luciferase reporter plasmid, which was named P5. Then, this plasmid was transfected into ME4405 cells expressing a wild-type p53 allele or p53 mutant alleles R175H or R273H. As shown in Fig. 5D, the luciferase activities of cells transfected with P5 significantly increased in response to the wild-type p53 but not to either mutant p53. Ecotopic expression of wild-type p53 and mutant p53 was verified by western blotting using a p53 antibody (Fig. 5E). To further clarify a critical role of this region in p53-mediated transcriptional activation of ITIH5, we transfected P5 into Mel-RM and Mel-CV cells with or without p53 knockdown. As shown in Fig. 5F and G, loss of p53 reduced the luciferase activities of cells transfected with P5.

To identify the p53-binding site on the ITIH5 promoter, we examined the sequence of P5 by the JASPAR database, and found a putative p53-binding site on the promoter region from −770 to −755 bp. To verify that this region was indeed responsive to p53, we constructed two pGL3-based luciferase reporter plasmids named BS for the wild-type-binding site and BSM for the mutant binding site (Fig. 5H). Then, these plasmids were individually transfected into 293 T cells with or without p53 overexpression. As shown in Fig. 5I, luciferase activities in cells transfected with the wild-type-binding site (but not the mutant binding site) was significantly elevated in response to p53. However, depletion of p53 decreased the luciferase activities of cells transfected with BS but not with BSM (Fig. 5J). Thus, these data indicated that the BS region was a functional binding site for p53 on the ITIH5 promoter.

To further verify that the BS region is indeed a functional p53-binding site on the ITIH5 promoter, we utilized ChIP assay with sequence-specific primers designed flanking the BS region showed that a chromatin fragment with the region of interest (P5). As shown in Fig. 5K, p53 bound to the P5 promoter region of ITIH5 as evidenced by the robust PCR amplification of DNA fragments in the anti-p53 immunoprecipitates of Mel-RM cells. Importantly, the binding capacity of p53 to the specific ITIH5 promoter region weakened when p53 was knocked down (Fig. 5L). Taken together, these findings indicate that p53 could bind to the specific promoter region (−770 to −755 bp) of ITIH5, thereby transcriptionally activating ITIH5.

### p53 inhibits the growth and migration of melanoma cells partly through ITIH5

**Fig. 6:**
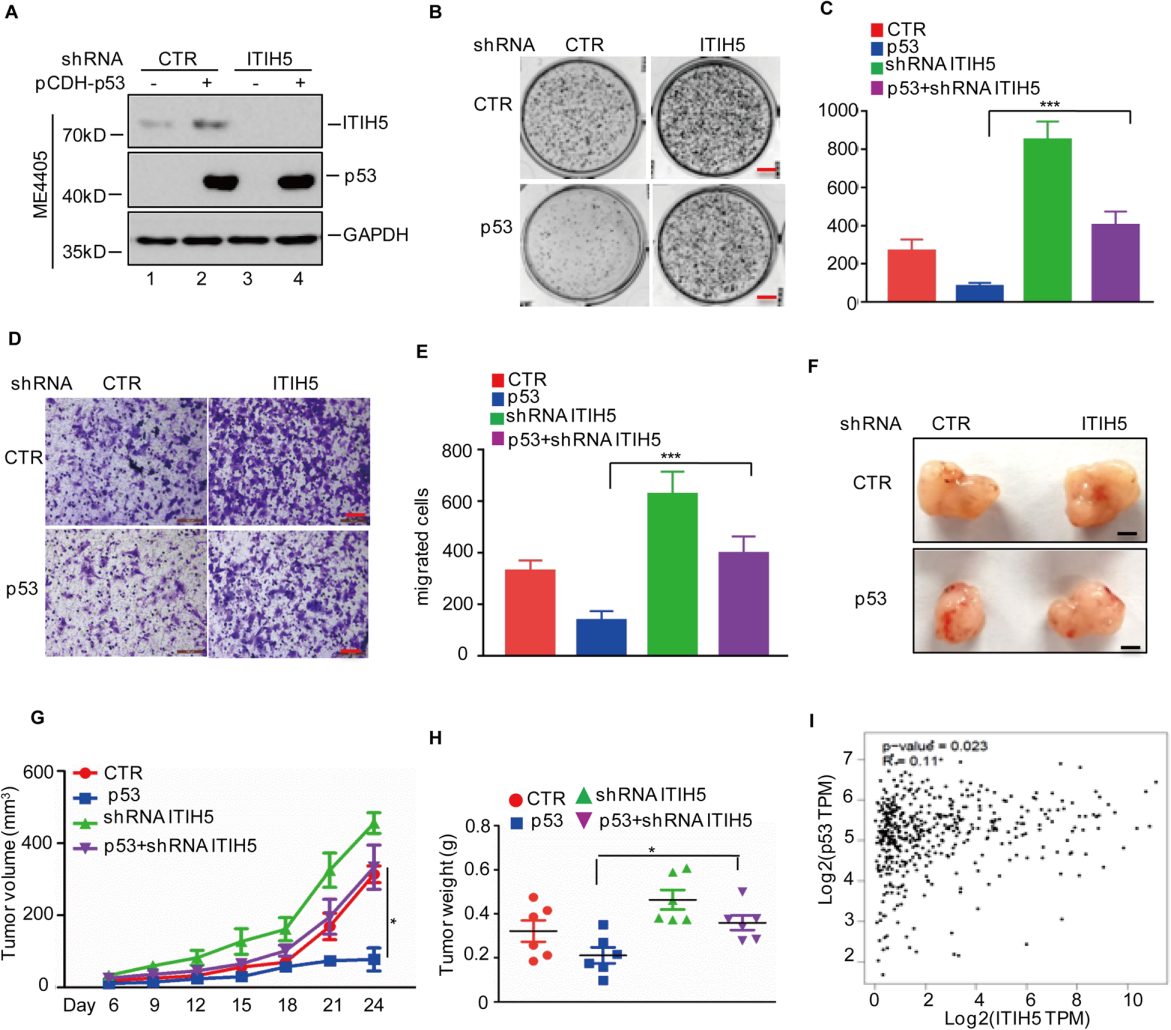
p53 suppresses melanoma cell growth and migration partly through ITIH5. **A** p53 was overexpressed in ME4405 cells with or without ITIH5 knockdown. The expression levels of ITIH5 and p53 were detected by western blotting. GAPDH was used as the loading control. **B**, **C** Representative images of culture plates (**B**) and average numbers of colonies (**C**) from the colony formation assays. Data in **C** represent three independent experiments, ***p < 0.001. Scale bar, 1 cm. **D**, **E** Represented images of crystal violet-stained culture plates (**D**) and average number of migrated cells (**E**) from Transwell cell migration assays. Data in **E** represent three independent experiments, ***p < 0.001. Scale bar, 100 μm. **F**–**H** The cells were injected subcutaneously (1 × 10^7^ cells per mouse) into nude mice (*n* = 6). Represented images of xenograft tumours (**F**). The volume (**G**) and weight (**H**) of the tumour were calculated. Scale bars: 0.5 cm. Data in **G**, **H** were analysed by Student’s *t*-test, *p < 0.05. **I** The association between ITIH5 and p53 was analysed in the GEPIA database.

To evaluate whether p53 inhibits the progression of melanoma by regulating the expression of ITIH5, we first depleted endogenous ITIH5 in the p53-overexpressing ME4405 cells and verified the predicted expression levels of ITIH5 and p53 in these cells by western blotting (Fig. 6A). We then assessed the alteration of cell proliferation by colony formation assays, and found that although increased p53 expression significantly inhibited cell growth, such inhibition was partially released when ITIH5 was knocked down (Fig. 6B). In Transwell cell migration assays, we also found that ITIH5 depletion partially reversed the decrease in cell migration by p53 (Fig. 6D, E).

To further confirm the functional consequence of ITIH5 depletion on the tumour-suppressive role of p53, we performed a xenograft tumour formation assay. We observed that depletion of ITIH5 partially reduced the inhibitory effect of p53 overexpression on tumour development (Fig. 6F–H). Furthermore, bioinformatic analysis of melanoma dataset from GEPIA database revealed a positive correlation between ITIH5 and p53 in melanoma (Fig. 6I). These results suggest that p53 depends at least in part on ITIH5 to inhibit tumour growth and metastasis in melanoma cells.

### ITIH5 interacts with KLF4

**Fig. 7:**
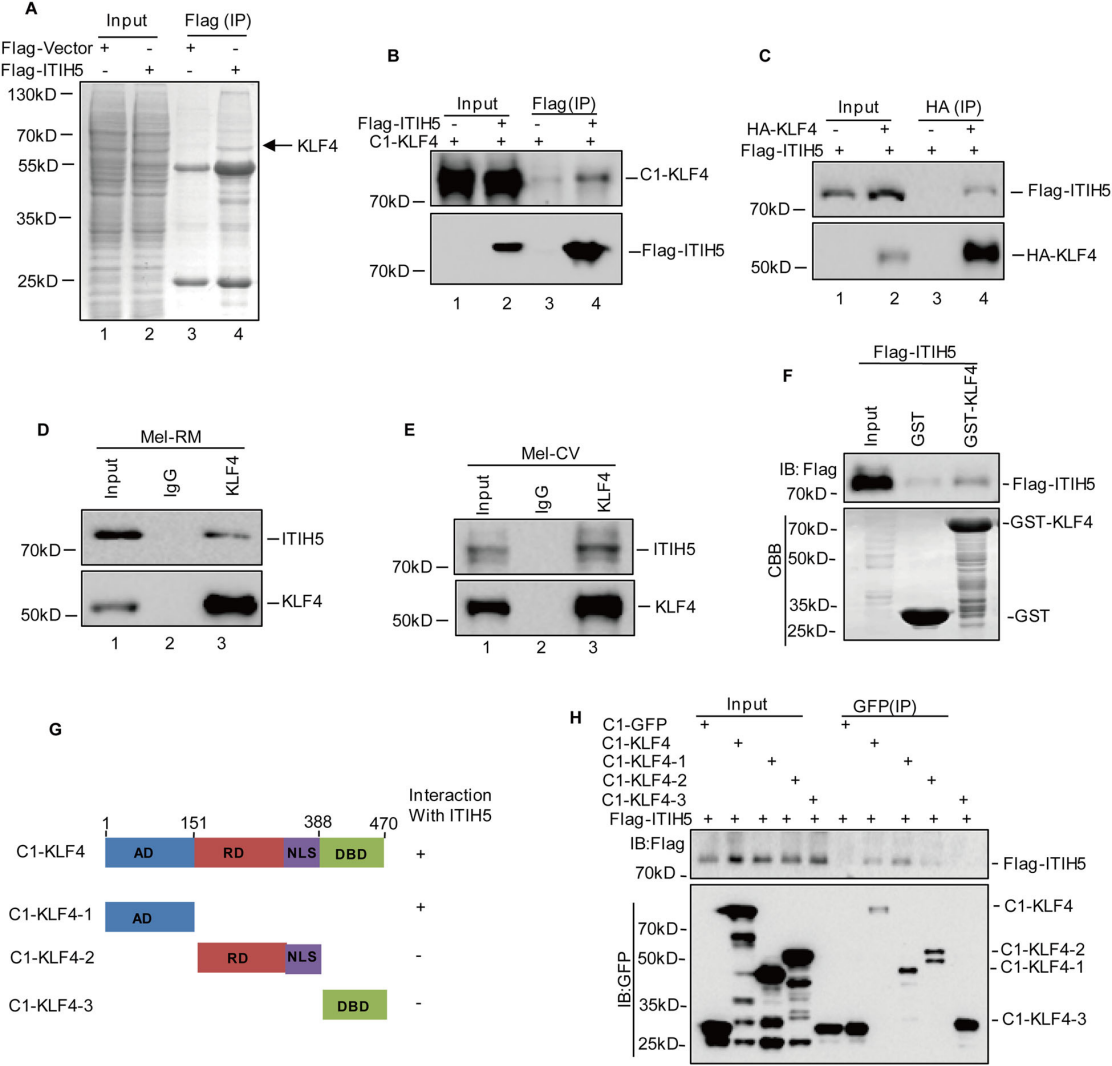
ITIH5 interacts with KLF4. **A** 293 T cell lysates with or without Flag-ITIH5 overexpression were incubated with Flag antibody. The binding proteins were eluted, resolved by SDS-PAGE and visualized by CBB staining. The arrowed band in the ITIH5 lane was identified as KLF4. **B**, **C** Flag-ITIH5 and C1-KLF4 were cotransfected into 293 T cells. Cell lysates were immunoprecipitated with anti-Flag or anti-GFP antibodies. **D**, **E** Lysates of Mel-RM and Mel-CV cells were immunoprecipitated with control IgG and anti-KLF4 antibodies. Immunoprecipitation was then detected with the indicated antibody. **F** Extracted and purified Flag-ITIH5 from 293 T cells was incubated with purified recombinant GST-KLF4 or GST at 4 °C for 12 h. Flag-ITIH5 retained on Sepharose was blotted with the Flag antibody. **G**, **H** Schematic diagram of the ITIH5 and KLF4 structure. AD activity domain, RD repression domain, NLS nuclear localization sequence, DBD DNA binding domain. The indicated constructs were transfected into 293 T cells. After 24 h, the cells were immunoprecipitated with anti-Flag antibody.

To uncover the molecular mechanism by which ITIH5 inhibits the tumorigenicity of melanoma cells, we sought to identify new ITIH5-interacting partners by mass spectrometry analysis. As shown in Fig. 7A, we found that KLF4 was a potential interacting protein of ITIH5. To confirm the physical association of ITIH5 with KLF4, we cotransfected Flag-ITIH5 and GFP-KLF4 into 293 T cells. A coimmunoprecipitation assay showed that GFP-tagged KLF4, which was expressed ectopically, could be detected in FLAG-tagged ITIH5 and vice versa (Fig. 7B, C). Importantly, an interaction between endogenous ITIH5 and KLF4 was also observed in Mel-RM and Mel-CV cells (Fig. 7D, E). Moreover, purified GST-KLF4, instead of the GST control, could bind to FLAG-tagged ITIH5 under cell-free conditions, suggesting a direct interaction between ITIH5 and KLF4 (Fig. 7F). To map the key binding domain of ITIH5 on KLF4, we expressed Flag-tagged ITIH5 along with different domains of KLF4 in HEK293T cells. A coimmunoprecipitation assay demonstrated that the transcriptional activity domain (AD) of KLF4 was essential for its physical interaction with ITIH5 (Fig. 7G, H). Collectively, these results validate the interaction between ITIH5 and KLF4.

### ITIH5 inhibits the progression of melanoma by downregulating the transcriptional activity of KLF4

**Fig. 8:**
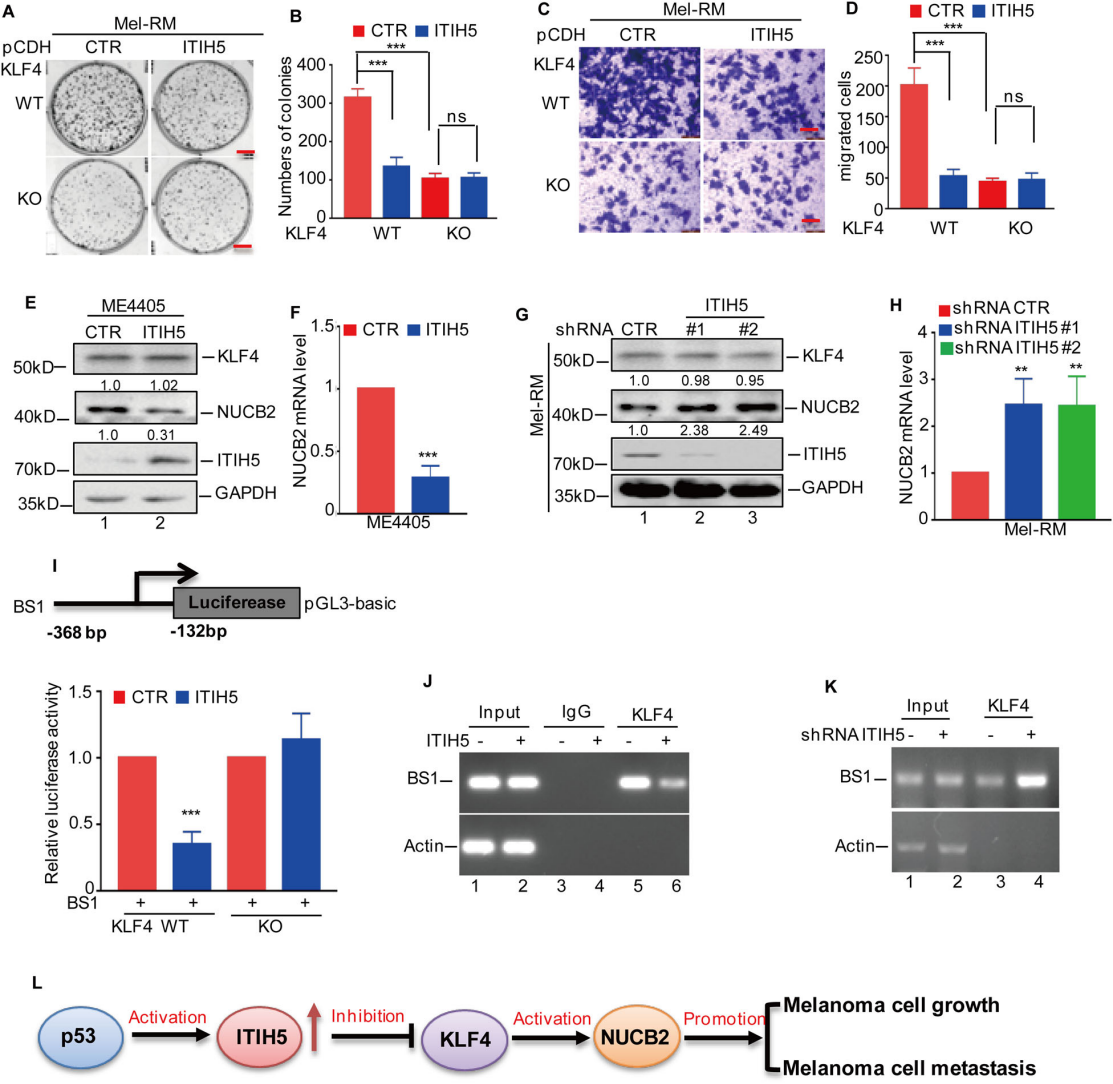
ITIH5 inhibits KLF4 transcriptional activity. **A**, **B** ITIH5 was overexpressed in Mel-RM cells with or without KLF4 knockout (KO). Representative images of culture plates (**A**) and average numbers of colonies (**B**) from the colony formation assays. Data in **B** represent three independent experiments, ***p < 0.001, ns not statistically significant. Scale bar, 1 cm. **C**, **D** Represented images of crystal violet-stained culture plates (**C**) and average number of migrated cells (**D**) from Transwell cell migration assays. Data in **D** represent three independent experiments, ***p < 0.001. Scale bar, 100 μm. **E**, **F** ITIH5 was overexpressed in ME4405 cells. The protein and mRNA levels of NUCB2 were analysed by western blotting (**E**) and RT-qPCR (**F**). GAPDH was used as the loading control. Data in **F** represent three independent experiments, ***p < 0.001. **G**, **H** ITIH5 expression was knocked down in Mel-RM cells. The protein and mRNA levels of NUCB2 were analysed by western blotting (**G**) and RT-qPCR (**H**). GAPDH was used as the loading control. Data in **H** represent three independent experiments, **p < 0.01. **I** Schematic illustration of pGL3-based reporter constructs used to assess the transcriptional activity of NUCB2 by luciferase assay. The promoters of NUCB2, P1 and ITIH5 were cotransfected into Mel-RM cells with or without KLF4 knockout (KO). The luciferase activity of P1 was measured. The results represent three independent experiments, ***p < 0.001. **J**, **K** ChIP analysis showed the binding of KLF4 to the promoter of NUCB2 in Mel-RM cells with or without ITIH5 overexpression (**J**) or knockdown (**K**). An isotype-matched IgG was used as a negative control. **L** Schematic diagram depicting the upregulation of ITIH5 expression by p53 that suppresses melanoma growth and migration through the negative regulation of KLF4 transcriptional activity.

Many studies have shown that KLF4 is an important oncogene in melanoma^24–26^. We therefore wanted to determine whether ITIH5 suppresses melanoma malignancy by downregulating KLF4. To this end, ITIH5 was overexpressed in Mel-RM cells with or without KLF4 knockout. We found that ITIH5 overexpression significantly suppressed cell proliferation and migration in KLF4 wild-type melanoma cells. However, the suppressive effects of ITIH5 overexpression on cell growth and migration were in the absence of KLF4 (Fig. 8A–D). These data suggest that inhibition of melanoma progression by ITIH5 depends on KLF4.

To understand the potential molecular mechanism of the suppressive effect of ITIH5 on KLF4-mediated melanoma progression, we first evaluated the protein levels of KLF4 in melanoma cells with or without ITIH5 knockdown or overexpression. As shown in Figs. 8E, G, we surprisingly found that the altered expression of ITIH5 did not affect the protein levels of KLF4. KLF4 is a well-known transcription factor that regulates tumorigenesis in a manner dependent on its transcriptional activity^27^. Based on this, we assessed the effect of ITIH5 on the expression of NUCB2, a known target gene of KLF4, in melanoma cells^24^. Compared with those of the control cells, the mRNA and protein levels of NUCB2 decreased when ITIH5 was overexpressed (Fig. 8E, F). In contrast, the expression of NUCB2 was significantly increased in the ITIH5-depleted cells (Fig. 8G, H).

To further explore whether ITIH5 directly represses the transcriptional activity of KLF4, leading to reductions in NUCB2, we cloned the region of the NUCB2 promoter that contained the KLF4-binding site. The results from luciferase reporter assays revealed that the activity of the NUCB2 promoter significantly decreased in the ITIH5-overexpressing cells. However, the effect of ITIH5 on NUCB2 promoter activity diminished when KLF4 was knocked out. Similarly, subsequent ChIP assays showed that the chromatin fragment enriched by KLF4 was reduced when ITIH5 was overexpressed (Fig. 8J). In addition, ITIH5 knockdown enhanced the binding capacity of KLF4 to the NUCB2 promoter (Fig. 8K). Collectively, these data indicate that ITIH5 inhibits the transcriptional activity of KLF4 even without affecting its protein levels.

## Discussion

In this study, we showed that ITIH5 expression was markedly reduced in melanoma tissues compared with normal skin tissues. Increased ITIH5 expression inhibited melanoma cell growth and metastasis in vitro and ex vivo. However, depletion of ITIH5 expression accelerated melanoma progression. Further mechanistic investigations revealed that in melanoma cells, p53 enhanced the expression of ITIH5 through transcriptional activation. Moreover, we also found that ITIH5 interacted with KLF4 to inhibit its transcriptional activity, resulting in downregulation of NUCB2, a known target gene of KLF4 (Fig. 8l).

ITIH5 is the fifth heavy chain member of the ITI family, and is considered to be an important ECM modulator^9^. Increasing evidence indicates that ITIH5 plays a key tumour-suppressive role in various cancers. For example, ITIH5 suppressed breast cancer metastasis and induced cell death by regulating TGF-β superfamily signalling switches, and elevated the expression of the tumour-suppressor gene DAPK1 by modulating epigenetic reprogramming^9,19,20^. In pancreatic cancer, overexpression of ITIH5 inhibited cell motility and invasion, and ITIH5 variant without secretion signal decreased pancreatic cancer liver metastasis^21,22^. In lung and bladder cancer cells, loss of ITIH5 was associated with unfavourable prognosis^17^. In extending the aforementioned findings, we present a couple of lines of evidence to support that ITIH5 plays a tumour-suppressive role in melanoma. First, the expression levels of ITIH5 were significantly downregulated in melanoma tissues compared with normal skin tissues. Loss of ITIH5 was associated with high metastasis and poor prognosis. Second, overexpression of ITIH5 suppressed melanoma metastasis and tumorigenesis in vitro and ex vivo.

Several studies have revealed that DNA hypermethylation is the main mechanism for the downregulation of ITIH5 in various cancers^14–16^. However, the transcription factors involved in regulating ITIH5 are still unclear. Here, our data showed that p53 directly bound to the promoter of ITIH5 and elevated its expression in melanoma cells. p53 is a key tumour suppressor that is activated under cellular stresses to prevent malignant transformation by activating a variety of biological processes, such as DNA repair, cell cycle arrest and cell apoptosis. The gene encoding p53 is known to frequently undergo gain-of-function or inactivating mutations in many human cancers^28,29^. However, in melanoma mutation of p53 is unusual, and wild-type p53 is often present^30,31^. Although p53 has been reported to facilitate the survival of melanoma cells through some cellular stress responses^32^, it has also been found to act as a tumour suppressor in melanoma^33^. In the present study, we revealed a new mechanism by which p53 suppressed the growth and metastasis of melanoma cells through transcriptional activation of ITIH5.

To reveal the potential molecular mechanism by which ITIH5 inhibits the tumour progression of melanoma, we used ITIH5 as bait to search for new ITIH5-interacting partners by mass spectrometry analysis. We found that KLF4 was an interacting protein of ITIH5. KLF4, a member of the Kruppel-like factor (KLF) subfamily of zinc-finger proteins, was reported to function as an oncogene in melanoma^25^. Substantial evidence indicated that KLF4 was not stable and was modulated by ubiquitylation^34,35^. Therefore, we first investigated whether ITIH5 could affect the expression of KLF4. Unexpectedly, we found that overexpression or knockdown of ITIH5 did not change KLF4 protein levels in melanoma. Our previous study showed that KLF4 promoted melanoma cell metastasis by transcriptionally regulating NUCB2 expression^24^. Thus, we investigated whether ITIH5 could regulate the transcriptional activity of KLF4 and affect NUCB2 expression. Interestingly, our data suggested that ITIH5 suppressed the transcriptional activity of KLF4 and prevented its binding to the NUCB2 promoter. In fact, inhibition of NUCB2 expression by ITIH5 relied on its interaction with KLF4 and blocked the binding of KLF4 to the NUCB2 promoter. However, the specific mechanism by which ITIH5 prevents the binding of KLF4 to the NUCB2 promoter is still unclear and will be investigated in the future.

In summary, our study identified a tumour-suppressive role of ITIH5 in melanoma, provided new insights into the regulatory mechanism of ITIH5, and revealed that ITIH5 was a novel transcriptional target of p53. In addition, our data demonstrated that ITIH5 was a bona fide interacting protein of KLF4 that suppressed the transcriptional activity of KLF4 and downregulated its target gene NUCB2, leading to the suppression of melanoma.
